# Mutation of the Mouse *Syce1* Gene Disrupts Synapsis and Suggests a Link between Synaptonemal Complex Structural Components and DNA Repair

**DOI:** 10.1371/journal.pgen.1000393

**Published:** 2009-02-27

**Authors:** Ewelina Bolcun-Filas, Robert Speed, Mary Taggart, Corinne Grey, Bernard de Massy, Ricardo Benavente, Howard J. Cooke

**Affiliations:** 1 MRC Human Genetics Unit and Institute of Genetics and Molecular Medicine, Western General Hospital, Edinburgh, United Kingdom; 2 Institute of Human Genetics, Centre National de la Recherche Scientifique, UPR1142, Montpellier, France; 3 Department of Cell and Developmental Biology, Biocenter of the University of Würzburg, Würzburg, Germany; National Cancer Institute, United States of America

## Abstract

In mammals, the synaptonemal complex is a structure required to complete crossover recombination. Although suggested by cytological work, *in vivo* links between the structural proteins of the synaptonemal complex and the proteins of the recombination process have not previously been made. The central element of the synaptonemal complex is traversed by DNA at sites of recombination and presents a logical place to look for interactions between these components. There are four known central element proteins, three of which have previously been mutated. Here, we complete the set by creating a null mutation in the *Syce1* gene in mouse. The resulting disruption of synapsis in these animals has allowed us to demonstrate a biochemical interaction between the structural protein SYCE2 and the repair protein RAD51. In normal meiosis, this interaction may be responsible for promoting homologous synapsis from sites of recombination.

## Author Summary

Production of sperm and eggs, also known as gametes, requires a reduction in the number of copies of the genome, from the two found in most cells of the body to the single copy found in gametes. This is a complex process, made even more complex because it is coupled with recombination, a process that is an important contributor to genetic diversity. Mammals and many other organisms achieve reduction and recombination through a process called meiosis, which is recognisable by the presence of a distinctive structure—the synaptonemal complex—that links the chromosomes together and is essential for meiosis to complete. We have made mice that lack SYCE1, a protein component of the synaptonemal complex. In these animals, meiosis is blocked at a particular stage, and this has allowed us to detect co-localisation and interactions—likely indirect—between enzymes involved in recombination and structural proteins involved in meiosis. This provides a starting point to understand in biochemical detail the protein links between structure and function in meiosis. Mutations or variants in the genes encoding such proteins are likely contributors to variations in fertility and to abnormalities in chromosome number.

## Introduction

Meiosis is a specialised process in which the replicated diploid genome undergoes two rounds of cell division without an intervening DNA replication. Production of haploid gametes from the diploid germ line is a complex process requiring the accurate separation of the two parental genomes to avoid the aneuploidy which would result from errors. Meiotic recombination imposes the additional requirement that the two genomes be precisely aligned for exchange of genetic information. In organisms from budding yeast to humans a key component of the meiotic cellular machinery used to enforce this is the synaptonemal complex (SC). This is a widely occurring, proteinaceous structure which physically links the pairs of sister chromatids (for review see [1]) and is visualised in the electron microscope as a zipper like structure with two lateral elements (LE) and the central element (CE) in between. Lateral elements are derived from axial elements (AE) that connect sister chromatids after premeiotic DNA replication. To date, numerous protein components of the SC have been defined in a variety of organisms (reviewed in [1]). They can be classified as components either of the LE/AE or of the CE. In mammals AE proteins include cohesins and coiled coil domain proteins such as SYCP3 and SYCP2 [2]–[4]. The CE contains the recently described proteins SYCE1, SYCE2 and TEX12 [5],[6]. SYCP1 is a key protein, which links AEs to the CE through its central coiled coil domain and by having C and N terminal globular domains anchored in AE and CE respectively [7]–[9]. In many organisms the formation of the SC is dependent on double strand breaks (DSBs) which can be processed to crossover or, more frequently, non crossover pathways. The SC may play a role in regulating the non random distribution of crossovers known as interference. However the requirement for and intact SC is sexually dimorphic in mice and it is not required for interference in female meiosis [10].

In male mice the fully assembled SC is required to complete crossover recombination and genetic exchange. Mutations in axial element components *Sycp2* and *Sycp3* result in failure of SC formation and infertility in the male. Milder meiotic defects in female meiosis result in increased aneuploidy and reduced litter sizes [11]–[13]. To date mutagenesis of known components of the CE in mouse suggest that an intact CE is required in both sexes. In *Sycp1* null mice synapsis is completely abolished and although the MSH4 foci indicative of intermediate stages of recombination are present neither sex forms the MLH1 foci, which are the cytological markers of crossover, and both sexes are infertile [14]. *Syce2* null mice, in which the axial elements align but do not synapse, also do not form MLH1 foci in either sex although again proteins indicative of earlier stages of the recombination process such as RAD51 and MSH4 are present [15]. TEX 12, a central element protein which interacts with SYCE2, has recently been shown to have a similar null phenotype with the absence of crossover recombination in both sexes [16]. Since these proteins are mutually dependent for localisation to and formation of the CE this similarity is not surprising.

Based on known interactions between SYCP1, SYCE1, SYCE2 and TEX12 (Figure S1) we have suggested that the assembly of the SC is a multi-step process which is blocked at different stages by the absence of SYCE1 and 2 and probably TEX12 [15]. In the presence of SYCE2 and the absence of SYCE1 the prediction is that points of synapsis, as observed in the *Syce2^−/−^* animals, do not occur. Here we report the phenotype of such mutant animals. Importantly this phenotype has suggested interactions between these structural components of the SC and the recombination machinery.

## Results

### Disruption and Inactivation of the Mouse *Syce1* Gene

We disrupted the mouse *Syce1* gene by gene targeting in AB2.2 ES cells. The targeting vector was designed to replace exons 2–11 of the *Syce1* gene with the *LacZ- Neo^r^* selection cassette (Figure S2A). Correct targeting was confirmed by Southern Blot analysis (Figure S2B). Correctly targeted ES cells were injected into C56BL/6 blastocysts and produced two germline transmitting chimeras. Offspring produced by mating these chimeras to C56BL/6 females were genotyped by PCR (Figure S2C) and *Syce1^+/tm1HGU^* animals intercrossed. Animals were produced from these matings with all genotypes in Mendelian ratios. To confirm the absence of the SYCE1 protein in the *Syce1^tm1HGU /tm1HGU^* (*Syce1^−/−^*) animals we used Western blotting. A polyclonal antibody raised against C-term of SYCE1 detects a protein band of the expected size (45 KDa) in wild-type testis extracts but not in the *Syce1^−/−^*, confirming the specificity of antibodies as well as indicating that the *Syce1* disruption described here results in a null mutation (Figure S2D). The lack of detectable proteins demonstrates the absence of splicing between the *Neo^r^* gene and remaining *Syce1* exons which might produce truncated proteins.

### Defects in Gametogenesis of the *Syce1*-Deficient Mice Confirm Its Role in Meiosis

*Syce1^−/−^* mice are infertile. Mating of both sexes with wild-type animals failed to yield any offspring although *Syce1^−/−^* males produced copulatory plugs suggesting normal sexual behaviour. *Syce1* mutant ovaries were minute and testes size was only 20–30% of wild-type littermates, which is similar to other meiotic mutants [12], [14]–[16]. We observed no phenotypes in other tissues of these animals.

**Figure 1:**
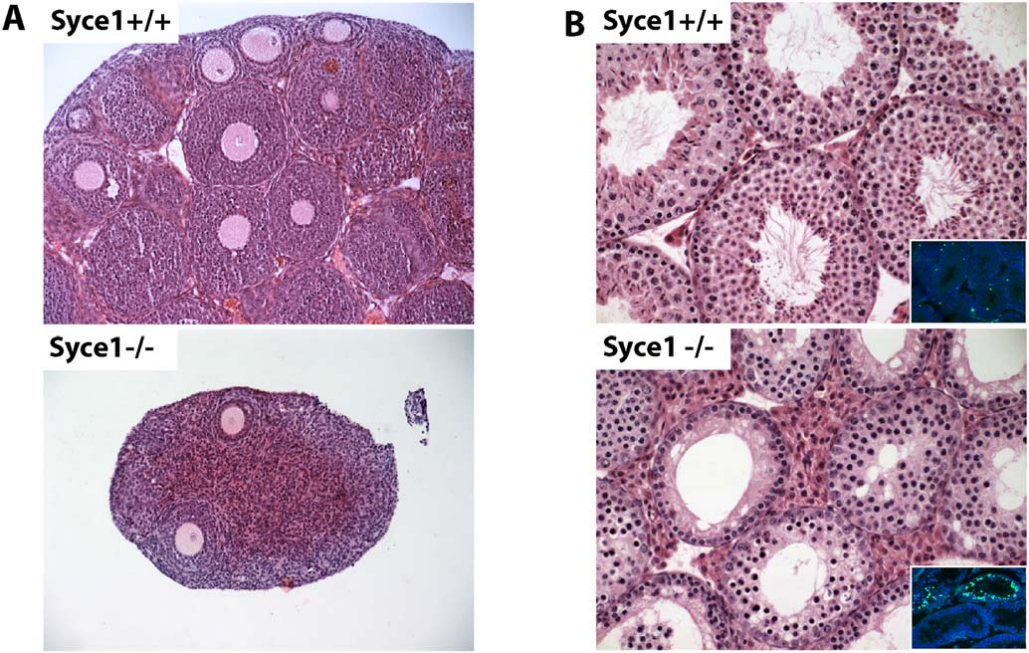
*Syce1* knockout animals show severe defects in gametogenesis. Haematoxylin and eosin (H&E) stained adult ovaries (A) and testes (B) from wild-type and *Syce1^−/−^* mice. (A) Mutant ovaries are greatly reduced in size and almost completely depleted of follicles in comparison to wild-type littermate with numerous follicles. (B) All stages of the spermatogenic cycle are apparent in the wild-type testis. *Syce1^−/−^* testis shows a reduced diameter of the seminiferous tubules and lack of postmeiotic stages. Insets, TUNEL assay for apoptotic cells. Occasional positive cells are present in the wild-type testis. In contrast, tubules with large number of positive cells were found in the *Syce1^−/−^* testis.

Histological analysis of adult *Syce1^−/−^* gonads revealed an almost complete lack of follicles in ovaries (Figure 1A), suggesting a disruption during meiosis followed by apoptosis, and lack of postmeiotic cells in the testis (Figure 1B). Primary spermatocytes were the most common germ cell type indicating a spermatogenesis arrest at prophase I. Elevated levels of apoptosis were detectable in some tubules by TUNEL staining (Figure 1B, insets) suggesting that arrested cells are eliminated by this mechanism. The high number of positive cells in a fraction of tubules indicates that most of the cells undergo apoptosis at the same epithelial stage, which was determined to be stage IV (data not shown). *Syce1^−/−^* females show a meiotic prophase phenotype similar to males indicating that SYCE1 plays the same role in both male and female meiosis. The lack of mature gametes is consistent with the expected role of SYCE1 protein in meiosis and demonstrates that *Syce1* is an essential gene for both male and female fertility.

### *Syce1* Mutant Spermatocytes Arrest during Pachynema due to Chromosome Synapsis Failure

**Figure 2:**
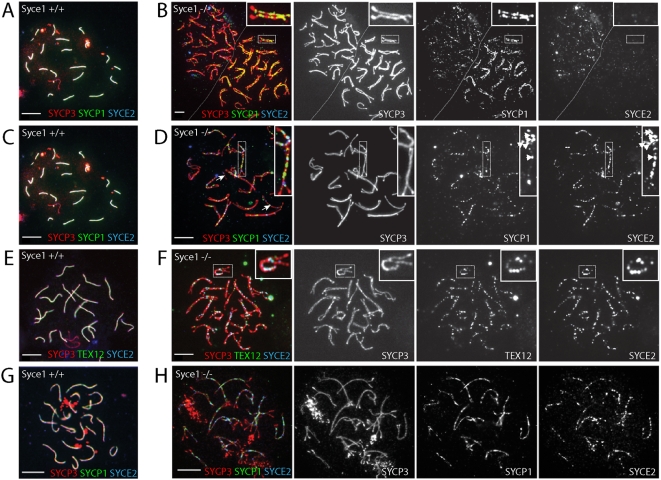
Homologous chromosomes fail to synapse in *Syce1^−/−^* mutant mice. Chromosome spread nuclei from wild-type and *Syce1^−/−^* spermatocytes (A–F) and oocytes (G–H) were immunostained with anti-SYCP3 to detect the AE and anti-SYCP1, anti-SYCE2 and anti-TEX12 for the CE. Wild-type cells show AEs fully formed and linked by the SC where SYCP1 and SYCE2 or TEX12 co-localise (A,C,E,G). In contrast, *Syce1^−/−^* spermatocytes and oocytes fail to form a complete SC between homologously aligned AEs (B,D,F,H). SYCP1 binds to aligned AEs in the absence of SYCE2 in (B), however the signal is weaker than in wild-type and discontinuous. (D,H) SYCP1 and SYCE2 localise to aligned AEs but do not always co-localise with each other as expected (D, inset). (F) SYCE2 and TEX12 co-localise in *Syce1^−/−^* spermatocytes (inset). Scale bar 10 µm.

To investigate the cause of the meiotic defect in more detail we prepared surface spread chromosomes from *Syce1^−/−^* spermatocytes. Normally during meiotic prophase I homologous chromosomes are closely juxtaposed and are then physically connected by the SC along the entire length of chromosome axes. Immunostaining for SYCP3, SYCP2 and STAG3 proteins revealed that AEs are formed normally in the absence of SYCE1 (Figure 2 and S3) and that homologous chromosomes align in close juxtaposition. The sex chromosomes are an exception to this; as in *Sycp1, Tex12 and Syce2* null mutants the pseudoautosomal regions do not pair and a sex body is not formed (Figure 2D, arrows). Wild-type spermatocytes at pachynema are characterised by the presence of ribbon-like structures seen by staining for SYCP1. These represent fully formed SCs linking homologous chromosomes (Figure 2A). In *Syce1^−/−^* cells, although AEs are formed and aligned SCs do not assemble between them as indicated by the absence of continuous SYCP1 staining (Figure 2B,D). Interestingly a weak discontinuous SYCP1 signal was observed associated with AE whether they are closely aligned or not (Figure 2B, D). We used immunostaining for SYCE2 and TEX12, two other markers of synapsis that in the wild-type co-localise with SYCP1 (Figure 2E) to further investigate synaptic failure. Although SYCE2 and TEX12 foci co-localise as expected, immunostaining for SYCE2 or TEX12 does not resemble that of the wild-type animals. Instead they were found in intermittent foci between closely aligned AEs (Figure 2F). This is consistent with the observations that their localisation to the SC is co-dependent and their known interactions (Figure S1) [6],[15],[16]. Unlike in wild-type spermatocytes, in *Syce1^−/−^* spermatocytes SYCE2 does not always follow SYCP1 signal either locally within a pair of homologs or globally in one nucleus (Figure 2D, B respectively). A subset of cells shows accumulation of SYCP1 on both AEs without accompanying SYCE2, suggesting that the SYCP1 C-terminal region can bind to AEs in the absence of SYCE1. Additionally in *Syce1/Syce2* double knockout SYCP1 still binds to aligned AEs suggesting that it is the presence of SYCE1 that restricts SYCP1 binding to synapsed axes when all components are present (not shown). *Syce1^−/−^* oocytes display very similar defects in chromosome synapsis to males (Figure 2G–H). AE are fully formed and homologous chromosomes align, however tripartite synaptonemal complex is not formed along the length of chromosomes.

**Figure 3:**
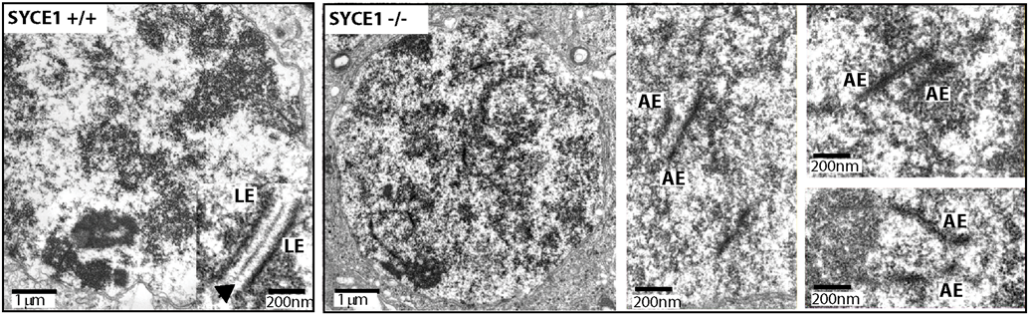
Electron Microscopy of the synaptonemal complex in wild-type and *Syce1^−/−^* spermatocytes. Left panel represents a wild-type cell with representative SC in the inset. The arrow indicates the electron dense CE. Right panels show mutant cells. Parallel AE were observed in *Syce1* mutant spermatocytes but SC with a CE was not found. LE- lateral elements, AE- axial elements, CE- central element.

In some cases AEs are in very close apposition along their length with spacing similar to that of the normal SC with SYCE2 and SYCP1 co-localised between them. In order to determine whether these sites of co-localisation of CE proteins represent SC formation we have performed electron microscopy on testis sections from *Syce1^−/−^* animals. Extensive analysis of the mutant material revealed presence of parallel AEs but failed to find any signs of the CE (Figure 3). This is in contrast to the *Syce2* or *Tex12* nulls, where CE-like structures were observed [15],[16]. Based on the observations from all three mutants we propose that the SYCE1 protein is required not only to stabilise SYCP1 dimers within central element but also to stack the transverse filaments into layers to form CE and determine the thickness of the SC.

### Meiotic DSB Are Formed but Are Not Efficiently Repaired in the Absence of SYCE1

**Figure 4:**
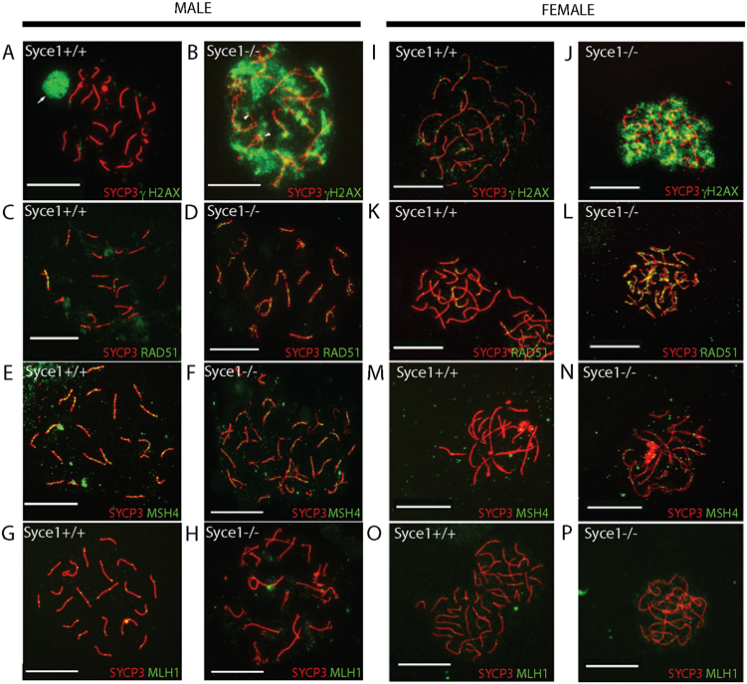
Meiotic DSB are generated but are not efficiently repaired and crossovers are not formed in the absence of SYCE1. Chromosome spreads from wild-type and *Syce1^−/−^* spermatocytes and oocytes were stained for known markers of meiotic recombination. (A,B,I,J) γH2AX marks sites of DSB, (C,D,K,L) RAD51 is a marker for early Recombination Nodules (RN), (E,F,M,N,) MSH4 is a marker of recombination intermediates and (G,H,O,P) MLH1 represents crossover sites. Scale bar 20 µm.

Meiotic recombination is initiated by SPO11-mediated double strand breaks (DSB) [17]. The generation and the repair of these breaks are required for chromosomal synapsis in most organisms including mammals [18]–[21]. The appearance of these breaks is accompanied by the phosphorylation of histone H2AX on large domains of chromatin around the break. As meiosis proceeds to the pachytene stage γH2AX is removed from synapsed chromosomes and is restricted to the largely asynapsed sex chromosomes in the XY body [22]–[24] (Figure 4A). *Syce1^−/−^* spermatocytes showed extensive γH2AX staining in early cells that persisted to the most advanced spermatocyte stages (Figure 4B)(in these animals the sex body does not form). Oocytes show the same pattern of staining (Figure 4J). This suggests that DSB are generated in the *Syce1^−/−^* mutants but are not efficiently repaired.

To assess the state of DSB repair in mutant spermatocytes and oocytes we analysed the distribution of proteins involved in different steps of meiotic repair and recombination [25],[26]. First the strand exchange proteins RAD51 and DMC1 are recruited to the sites of DSB and form early recombination nodules (EN). RAD51/DMC1 mediate the homology search and the single end invasion of the homologous chromosome [27]. Cytologically, RAD51 and DMC1 manifest as numerous foci along chromosome cores, typically several hundred occur in a mouse meiotic nucleus [28]. During normal meiosis numbers of RAD51/DMC1 foci peak at leptonema and disappear by mid-pachynema except along asynapsed cores of sex chromosomes in males (Figure 4C and K). RAD51 foci are highly abundant in both *Syce1^−/−^* spermatocytes and oocytes and are localised to both aligned and unaligned chromosome cores (Figure 4D and L). Fifteen percent of cells lack RAD51 foci entirely.

The *MutS* homologs MSH4 and MSH5 have been proposed to function in stabilization or resolution of recombination intermediates and possibly also during synapsis in earlier stages of prophase I [29]–[31]. In normal meiosis MSH4 foci appear concurrently with synapsis at early zygotene, peaking at late zygotene and starting to decrease at early pachytene (Figure 4E and M). In *Syce1^−/−^* spermatocytes and oocytes MSH4 foci appear without synapsis and are found only between aligned chromosome cores (Figure 4F and N). This indicates that MSH4/MSH5 mediated DNA-DNA interactions between homologous chromosomes can occur in the absence of SYCE1. Spermatocytes of mice lacking other proteins such as SYCP1 and SYCE2 which are required for synapsis also have MSH4 foci.

After *MutS* homologs MSH4/MSH5 associate with DNA a complex of *MutL* homologs MLH1/MLH3 is recruited to sites now termed late recombination nodules (RN). Together they are implicated in the processing of DSB through the double Holliday junction (dHJ) recombination intermediates that result in crossover. *Mlh1* was shown to be essential for crossover formation in mammals and yeast [32]–[34]. In wild-type meiosis MLH1 appears at late prophase in pachytene and is present in a few sites that correspond in number and distribution to the number of crossover events estimated genetically [35](Figure 4G and O). We stained *Syce1^−/−^* spermatocytes and oocytes with an anti-MLH1 antibody and failed to observe any MLH1 foci (Figure 4H and P). This indicates that despite MSH4 associated recombination intermediates MLH1 can not be recruited to resolve them into crossover in the absence of SYCE1 and synapsis or that cell death occurs before that stage.

Taken together, analysis of the progress of meiotic recombination suggests that SYCE1 is dispensable for the initiation of recombination but is essential for stable homologue interactions mediated by the SC and crossover formation.

### The *Syce1^−/−^* Phenotype Suggests a Link between Synaptonemal Complex and Early Recombination Proteins in Mouse

Recombination and synapsis are co-dependent and physically linked in yeast where synapsis is initiated at sites of recombination destined to be crossovers [36],[37]. To our knowledge no such link has been described in the mouse.

**Figure 5:**
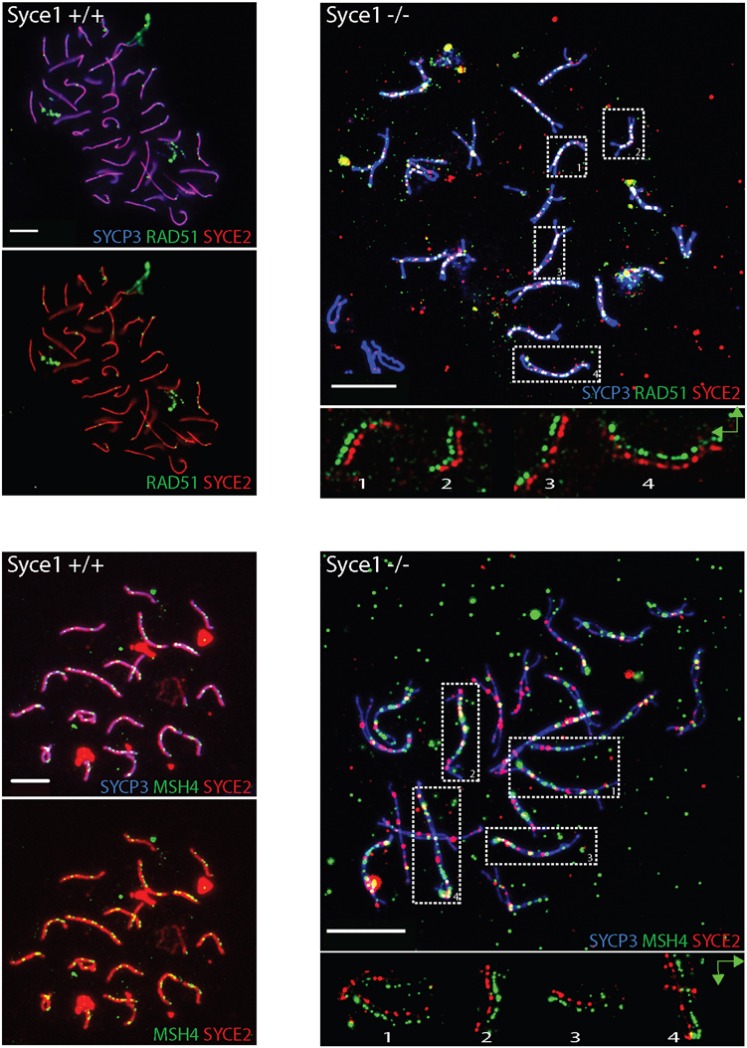
The *Syce1^−/−^* phenotype suggests a link between synapsis and recombination. Wild-type (A,C) and *Syce1^−/−^* (B,D) spermatocyte spreads immunostained with anti-SYCP3 for AE, anti-RAD51 and anti-MSH4 for recombination nodules and SYCE2 for the CE. (B) In *Syce1^−/−^* spermatocytes SYCE2 and RAD51 co-localise. (D) *Syce1^−/−^* spermatocyte showing partial co-localisation between SYCE2 and MSH4. Protein co-localisation on selected bivalents (1–4) shown with signals offset in the lower panels of B and D. Scale bar 10 µm.

**Figure 6:**
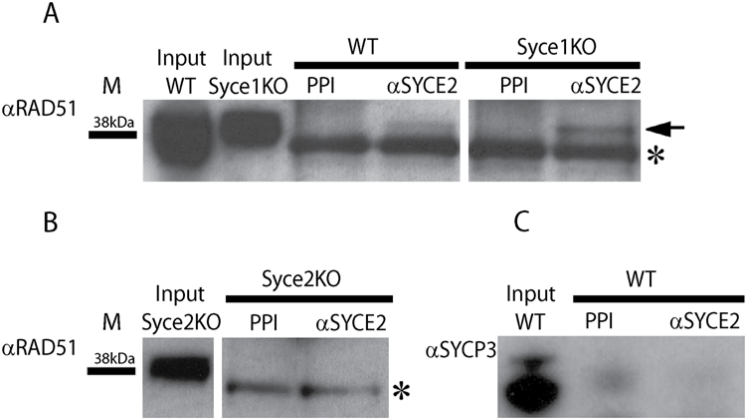
SYCE2 and RAD51 interaction detected by immunoprecipitation (IP) of testis extracts. (A) Extracts (0.5 mg–1.5 ml) from adult wild-type and Syce1^−/−^ testes were incubated with pre-immune serum (PPI- 5 µl) and anti-SYCE2 antibodies (10 µl) and precipitates (30 µl) analysed by Western blotting using an anti-RAD51 antibody. Inputs (10 µl) and IP samples (15 µl) were run in the same gel. RAD51 (arrow) was detected in samples precipitated with anti-SYCE2 antibodies but not with PPI. Asterisk- unspecific band. (B) Similarly, extracts from Syce2^−/−^ testes were immunoprecipitated with PPI and anti-SYCE2 antibodies and showed no signal for RAD51. (C) As an additional control wild-type extracts were immunoprecipitated in similar IP experiment and probed with anti-SYCP3 antibody. Absence of SYCP3 signal in this control excludes the possibility that the interaction observed in (A) is due to precipitation of the whole SC. M- Marker band corresponding to 38 kDa.

In *Syce1^−/−^* spermatocytes we noticed that the pattern of SYCE2/TEX12 foci between closely juxtaposed AEs resembles that of RAD51. To confirm our observations we immunostained *Syce1^−/−^* spermatocytes with anti-SYCE2 and -RAD51 antibodies. A subset of cells (42%, n = 435) with high number of RAD51 foci (approximately two hundred per nucleus) did not have any SYCE2 staining (Figure S4) However, cells with approximately half the number of RAD51 foci, located between aligned AE, showed co-localised staining for SYCE2 (43% n = 435) (Figure 5B). SYCE2 was almost always accompanied by a RAD51 signal in these cells (Figure 5B, lower panel in offset). To test if this co-localisation reflects a biochemical interaction between SYCE2 and RAD51 we used immunoprecipitation (IP) from wild-type and *Syce1^−/−^* testicular extracts. We have immunoprecipitated proteins using both anti-SYCE2 antibody and preimmune serum as a control, and checked for interacting proteins by probing western blot with anti-RAD51 antibodies. We were able to detect RAD51 as a band of approximately 37 KDa in the input as well as weakly in the wild-type and *Syce1^−/−^* IP samples but not in the control (Figure 6A). As a further control we have used *Syce2^−/−^* testis extract for IP with anti-SYCE2 antibodies and failed to detect RAD51(Figure 6B). To check if this interaction is specific and not due to the precipitation of the whole SC we tested SYCE2 IP samples with antiSYCP3 antibodies and did not detect SYCP3 in the immunoprecipitated sample (Figure 6C). Although we detect SYCE2 and RAD51 in the same complex we can not and do not conclude that this interaction is direct. Our attempts to demonstrate that using an in vitro assay have been inconclusive due to insolubility of proteins when co-overexpressed or to RAD51-GST interactions in pull down reactions. We proceeded to check if SYCE2 also co-localises with MSH4 which appears when chromosomes synapse and which succeeds RAD51 in the recombination nodules. Co-immunostaining of *Syce1^−/−^* spermatocytes for SYCE2 and MSH4 revealed that these two proteins only partially co-localise. (Figure 5D, and inset). There are different classes of cells: one which has only SYCE2 signals and no MSH4 (7.5%, n = 189, not shown), another which stains for both (36%, n = 189) (Figure 5D) and the remaining largest group shows only MSH4 foci (50%, n = 189) (Figure S4). This would suggest that as RAD51 is displaced by MSH4, SYCE2 is no longer associated with chromosomes in the *Syce1^−/−^* animals. Altogether, this data suggests that central clement protein SYCE2 interacts, directly or indirectly, with the recombination protein RAD51.

Is synapsis dependent on the RAD51/SYCE2 interaction? *Spo11* null mice are unable to generate meiotic DSB and as a result RAD51 is absent from the nucleus. Despite this, various degrees of synapsis, mostly nonhomologous, were observed in the *Spo11* null, on the basis of SYCP1 staining [20],[21]. We have stained *Spo11^−/−^* spermatocytes for SYCE1 and SYCE2 to check if these proteins are components of this DSB independent synapsis. Our results show that both SYCE1 and SYCE2 co-localise with SYCP1 on the SC in the *Spo11* mutants indicating that apparently normal synapsis can form in the absence of RAD51 and DSB (Figure S5), but in this case between random chromosomes.

## Discussion

Successful completion of meiosis in mouse depends on the assembly of the SC. Recent work using targeted mutagenesis to make null mutations in three (*Sycp1*, *Syce2* and *Tex12*) of the four known protein components of the CE has shown that the CE is a critical component of this structure [14]–[16]. Here we complete the set by mutating the remaining known component SYCE1. As predicted from the known multiple interactions of the proteins (Figure S1) *Syce1^−/−^* animals have a phenotype which is very similar to that of the other three null mutations. DNA repair is incomplete, the SC and the sex body are absent, homologous alignments at variable distances of the AEs occur, early (RAD51) and intermediate (MSH4) markers of recombination are present but there is a complete absence of MLH1 marking crossovers. In the testis cells are eliminated by apoptosis and both sexes are infertile. Complete assembly of the SC is co-dependent on the presence of all four proteins (SYCP1, SYCE1, SYCE2 and TEX12) and perhaps on others as yet undiscovered. However the mice null for different CE components are likely blocked in different states of SC assembly and provide tools to dissect this essential process.

There are distinct features of the *Syce1^−/−^* phenotype. In the absence of SYCE1 transverse filament protein SYCP1 binds to AEs when they are closely aligned and presumably forms N-termini associations [9]. This may reflect the protein's ability to form polycomplexes with dimensions corresponding to SCs [38]. However SYCP1 is also associated with AEs that are further apart confirming the proposal in our model that SYCP1 N-terminal associations alone are insufficient to promote SC assembly and require SYCE1 for stability in physiological conditions. The extensive association of SYCP1 with AEs in the *Syce1^−/−^* animals suggests that SYCE1 could play a role in restricting SYCP1 binding in wild-type synapsis. These associations with unpaired AEs are absent in the *Syce2^−/−^* and *Tex12^−/−^* males where SYCE1 is present [15],[16].

The *Syce1^−/−^* phenotype further supports the idea that SYCE2 and TEX12 act in concert. From published data we know that their localisation to the SC is co-dependent [15],[16] and in the absence of SYCE1 (this paper) both SYCE2 and TEX12 co-localise as foci between aligned AEs, therefore their recruitment to chromosome axes is SYCE1 independent. Previously, in our model for synaptonemal complex assembly we suggested that SYCE1 stabilises N-terminal interactions of SYCP1 in the CE and that SYCE2/TEX12 is required for the elongation of the SC. The *Syce1^−/−^* phenotype is consistent with this model.

Given the presence of three out of four CE components and interactions between SYCP1 and SYCE2 we expected some form of CE to be present in *Syce1^−/−^* spermatocytes as found in *Syce2^−/−^* and *Tex12^−/−^* spermatocytes. Our extensive analysis of testis sections at the EM level failed to detect a CE. Our model for CE assembly was two dimensional, reflecting observations in the light microscope and in EM sections but the SC has a thickness which we had not taken into account and of which SYCE1 may be a component [39]. In a revised model although the three CE proteins (SYCP1, SYCE2 and TEX12) co-localise they do not produce a visible CE in the microscope due to the absence of multiple layers of proteins dependent on SYCE1. We propose that SYCE1 stabilises the N-termini associations of SYCP1 (width) and regulates formation of transverse filament stacking (thickness) in addition to being required for SC extension through its interactions with SYCE2 and SYCP1.

Studies of the SC functions in various organisms revealed that the SC is essential for normal progression of meiotic recombination and formation of crossovers in yeast, plants and mammals [14],[40],[41]. It has been also shown that proper assembly of the SC between homologous chromosomes depends on recombination. In the absence of the SPO11 induced DSBs that initiate recombination, levels of SC formation are highly reduced or form between nonhomologous chromosomes [20],[21]. Additionally, the correct processing of DSBs at the early stages of recombination is essential for synapsis to occur [29],[31],[42],[43]. Impaired recombination in mouse mutants lacking the CE points to the possibility that interactions between the structural components of the CE and the recombination machinery occur and are essential for crossover. Prior to synapsis the recombinase RAD51 is recruited to the DSBs and disappears as chromosomes synapse. In mutants that lack the SC RAD51 persists longer and is associated with the AEs. It is not possible to study the function of RAD51 in meiosis due to embryonic lethality of the *Rad51* mutation [44]. However, the phenotypes of recently reported mutations in the *Tex15* and *Tex11 (Zip4H)* genes show that both recruitment as well as timely disappearance of RAD51 are crucial for synapsis and meiotic recombination. In the *Tex15* mutant RAD51 foci are highly reduced in number whereas in the *Tex11 (Zip4H)* mutant the number of these foci increases, probably as a result of delayed processing of DSB. Both mutants show synapsis defects. In *Tex11* null some chromosomes do not synapse at all and in *Tex15^−/−^* spermatocytes synapsis is completely abolished. As a result the number of MLH1 foci present in spermatocytes is reduced or eliminated, respectively [45]–[47]. In wild-type meiosis several different types of structures containing recombination proteins have been described based on immuno-histochemsitry. In leptotene RAD51/DMC1 foci have been termed early nodules (EN), later they begin to contain RPA in addition to RAD51/DMC1 and when synapsis is complete RAD51 is absent in RPA containing transition nodules (TN). The MLH1 containing recombination nodules (RN) appear last [26].

Based on our observation that SYCE2 and RAD51 co-localise in a subset of the *Syce1^−/−^* spermatocytes and that interactions between these proteins can be detected in testis extracts we propose that this interaction promotes synaptonemal complex assembly/extension. From a yeast two hybrid assay and in vitro pull down experiments it was previously suggested that SYCP1 interacts with RAD51 but not with DMC1 [48]. SYCP1 was also shown to recruit SYCE1 and SYCE2 to the SC as these proteins are not chromosomally localised in *Sycp1^−/−^* spermatocytes [5],[6] and hence must be involved in the RAD51/SYCE2 interaction. Although all four CE proteins are needed for complete synapsis, structures suggestive of sites of initiation of synapsis can be seen at both light and electron microscope resolution in the absence of SYCE2 or TEX12 but not in the absence of SYCE1. In the SYCE1 null animals we observe co-localisation of SYCE2 and RAD51 which we suggest occurs in normal mouse meiosis but is obscured by the subsequent rapid assembly of the SC. This concentration of SYCE2 may function to promote SC extension. We can not exclude that TEX12, a SYCE2 binding partner, plays a specific role in its interaction with RAD51. Interestingly, it was shown that in DSB deficient mutants, when breaks are introduced artificially, the number of RAD51 foci representing induced DSB correlate with the extent of synapsis [49]. This also points out the link between RAD51 and synapsis. However, it seems that RAD51 is not required in *Spo11* mutants for initiation and partial assembly of the SC [20],[21] but in these animals the SC is not formed between homologous chromosomes. Perhaps the presence of RAD51 at the sites of DSB favours the extension of homologous SC assembly over that of non homologous SC in a competitive and (in terms of aneuploidy) potentially disastrous situation.

Feedback from SC assembly must be required for the maturation of a small set of TN into the RN marking sites of recombination. The combination of cytology and enzymology has pointed to the ability of cellular structures to recruit and perhaps modify the function of repair enzymes for use in meiosis [50]. Our results here suggest that this process may also operate in the reverse direction with repair proteins playing a role in the assembly of structures essential for meiosis and fertility.

## Materials and Methods

### Generation and Characterisation of SYCE1-Deficient Mice

To inactivate the *Syce1* gene, we designed a targeting vector to replace exons 2–11 by selection cassette. This construct was based on a modified pBluescript vector containing *DTA* cassette, *En2SA-IRES-LacZ-pA* and floxed *tk-NEO* gene. A 5.2 kb *ApaI* fragment containing part of intron 1 of the mouse *Syce1* gene was cloned between *DTA* and *LacZ-Neo* cassettes and a 2.2 kb *SacI* fragment containing exons 12–13 of the *Syce1* gene was cloned downstream of *Neo* cassette. The linearised *Syce1* targeting construct was electroporated to AB2.2 ES cells. After selection with G418 ES cell clones were screened by PCR (FP: CAACCTCCCTCACCACCTTA, RP: TTGCTGAAGTTGTGCCAGAC). Potential positive clones were expanded and DNA was extracted for Southern blot analysis. DNA was digested with *EcoRI* and hybridised with external probe (See Figure S2). Cells from one of the correctly targeted ES clones were injected into C57/B6 blastocysts to obtain chimeras. Chimeric males were mated to C57/B6 females and progeny was genotyped using primers (FP:CCAGAAGCCTGAACATCTGACA, RP:TACCATCCTCCATGAGCTGTCT, Neo:AGGACATAGCGTTGGCTACCC). To produce *Syce1ko* mice we intercrossed heterozygous offspring. Tissues for histological examinations were dissected and fixed in Bouin's fixative. Subsequently, tissues were embedded in paraffin and 6 µm sections were cut. Mounted sections were deparaffinised, rehydrated, and stained with hematoxylin and eosin. Apoptosis was assayed using DeadEnd Fluorometric TUNEL System (Promega) according to the manufacturer's protocol

### Chromosome Spread Preparation and Immunostaining

Spread chromosomes from males and females were prepared and stained as previously described [5], Images were captured using a system comprising a charge-coupled device camera (Orca-AG; Hamamatsu), a fluorescence microscope (Axioplan II; Carl Zeiss MicroImaging, Inc.) with Plan-neofluar objectives (100× NA 1.3), a 100-W Hg source (Carl Zeiss MicroImaging, Inc.), and quadruple band-pass filter set (model 86000; Chroma Technology Corp.), with the single excitation and emission filters installed in motorised filter wheels (Prior Scientific Instruments). Image capture was performed using in-house scripts written for IPLab Spectrum (Scanalytics). Images were processed using Adobe Photoshop.

Electron microscopy was performed using ultra thin sections of testis tissue fixed in 2.5% glutaraldehyde and 1% OsO_4_ as described previously [51].

The primary antibodies used were: rabbit anti-SYCE1; rabbit anti-SYCE2 [5]; guinea pig anti-SYCE1; guinea pig anti-SYCE2; guinea pig anti-TEX12 [6]; rabbit anti-SYCP1 (Abcam); mouse anti-SYCP3 [52]; rabbit anti-SYCP3 (Abcam); rabbit anti-STAG3 [53]; rabbit anti-SYCP2 [54]; rabbit anti-γH2AX (Upstate Biotechnology); mouse anti-Rad51 (Upstate Biotechnology); mouse anti-MLH1 (BD Biosciences); rabbit anti-Msh4 (Abcam). Secondary antibodies used were Alexa Dyes (AlexaFluor-488, 594 and 647) conjugates (Molecular Probes).

### Biochemical interactions

Protein extraction, immunoprecipitation and detection were carried out as previously described [5]

## Supporting Information

Figure S1 Network of CE protein interactions. Overlapping circles represent self interactions.
(0.3 MB TIF)

Figure S2 Targeted inactivation of the mouse *Syce1* gene. (A) Schematic diagram of the *Syce1* targeting strategy. Exons 2–11 (grey boxes) were replaced by *LacZ-Neo^r^* selection cassette. Genotyping primers are marked by arrows (B) Southern blot analysis of DNA digested with *EcoRI* and hybridised with external probe (see A). A wild-type band of 11 kb is detected in the control and two bands 11 kb wild-type allele and 7.5 kb mutant allele in three clones, indicating correct targeting. (C) PCR genotyping using primers shown in (A). (D) Western blot analysis of testis cell extracts from wild-type and *Syce1^−/−^* mice. The blot was probed with anti-SYCE1 antibody. A protein of the correct size was detected only in the wild-type extract. Abbreviations: A - *ApaI*; E -*EcoRI*; S - *SacI*; Ex.Pr.- External Probe.
(0.5 MB TIF)

Figure S3 *Syce1* mutant mice form normal AEs that align homologously. Surface-spread nuclei of wild-type and mutant meiotic cells were immunostained with antibodies against SC components SYCP2 and SYCP3 and cohesin STAG3. Scale bar 10 µm.
(1.1 MB TIF)

Figure S4 Immunostaining of representative *Syce1^−/−^* cells positive for RAD51 or MSH4 but lacking SYCE2 signal. Scale bar 10 µm.
(1.5 MB TIF)

Figure S5 Central Element proteins SYCE1 and SYCE2 are present in the nonhomologous SC in the *Spo11^−/−^* spermatocytes. Scale bar 10 µm.
(0.7 MB AI)
