# Correction: Mutation of the Mouse *Syce1* Gene Disrupts Synapsis and Suggests a Link between Synaptonemal Complex Structural Components and DNA Repair

**DOI:** 10.1371/annotation/50260271-aed9-4316-b09a-304591b0cba5

**Published:** 2009-04-03

**Authors:** Ewelina Bolcun-Filas, Emma Hall, Robert Speed, Mary Taggart, Corinne Grey, Bernard de Massy, Ricardo Benavente, Howard J. Cooke

An author was omitted from the published paper. Emma Hall should be listed as second author, affiliated with: MRC Human Genetics Unit and Institute of Genetics and Molecular Medicine, Western General Hospital, Edinburgh, United Kingdom. Ms. Hall's contribution to the paper was: contributed and analysed data.

