# Supplementary figures and images for: Mutation of the Mouse Syce1 Gene Disrupts Synapsis and Suggests a Link between Synaptonemal Complex Structural Components and DNA Repair
Source: PLoS Genet. 2009 Feb 27;5(2):e1000393. doi: 10.1371/journal.pgen.1000393 (PMC2640461; doi:10.1371/journal.pgen.1000393)

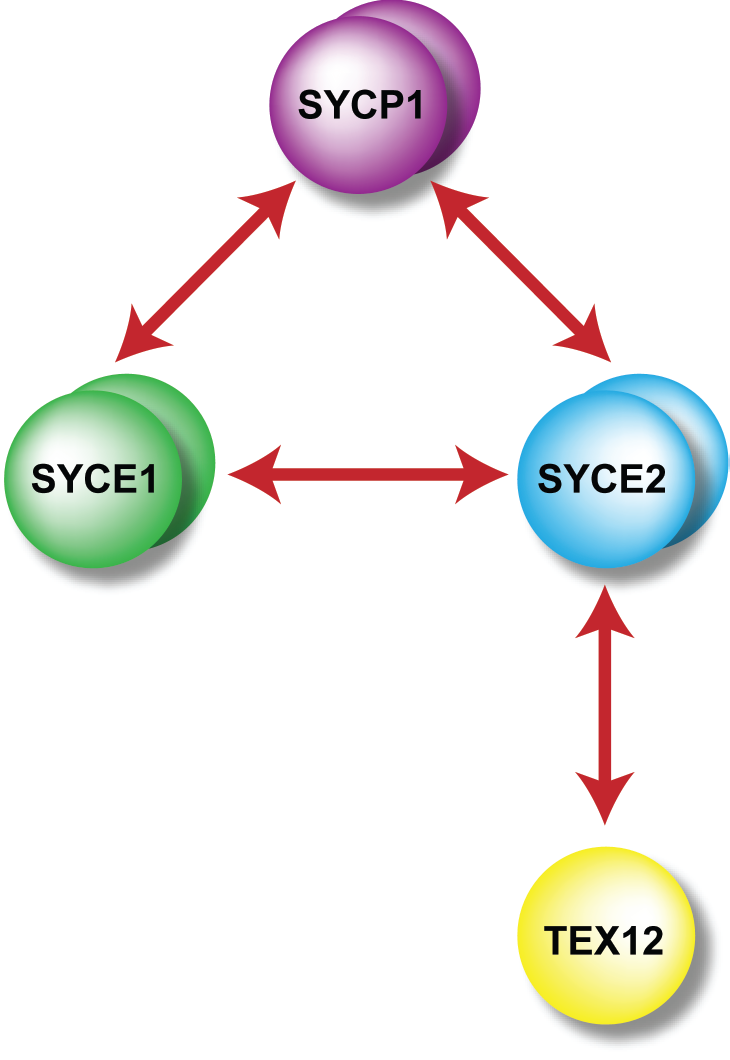

Supplement: Figure S1 — Network of CE protein interactions. Overlapping circles represent self interactions. (0.3 MB TIF) [file pgen.1000393.s001.tif]

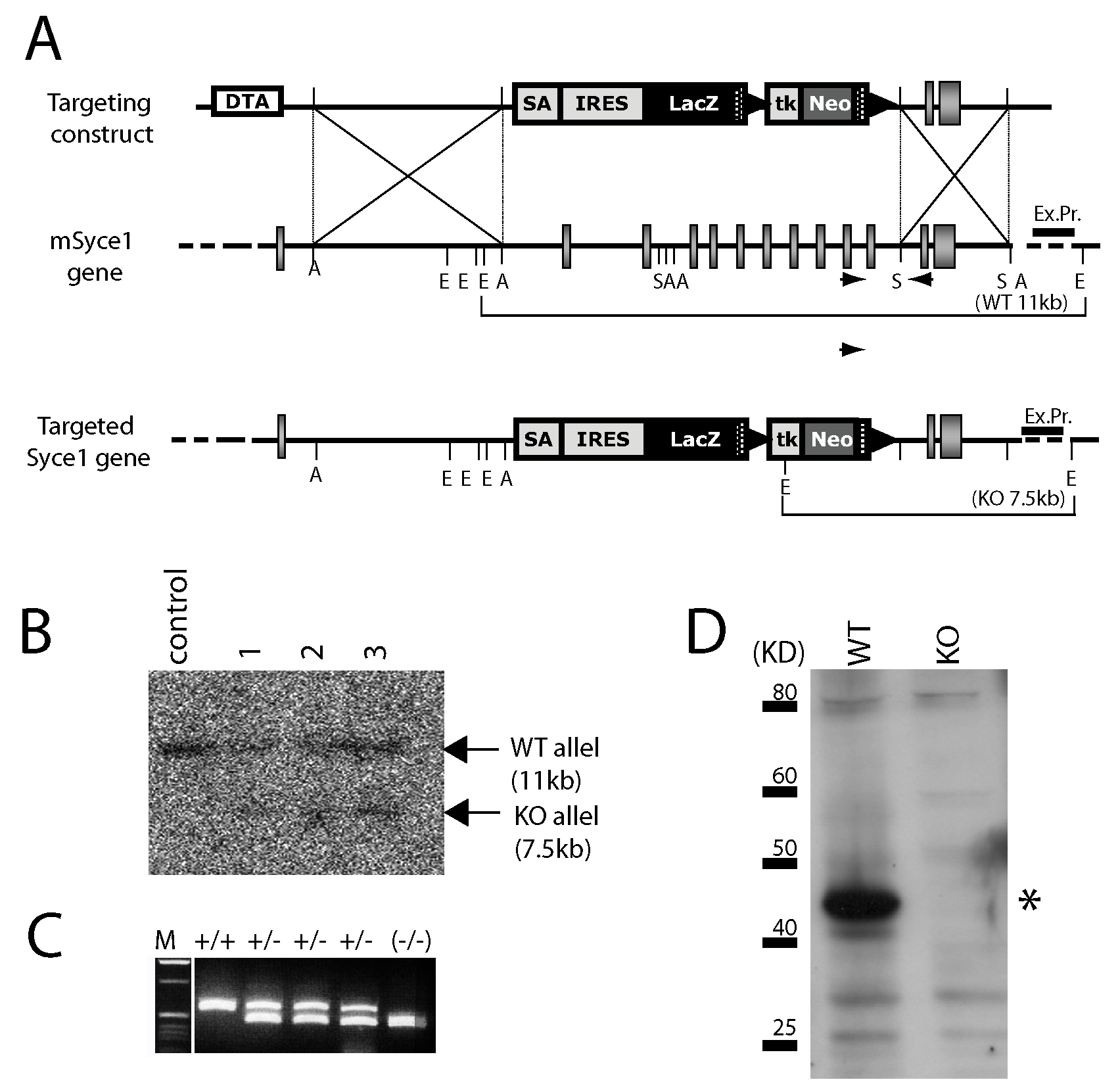

Supplement: Figure S2 — Targeted inactivation of the mouse Syce1 gene. (A) Schematic diagram of the Syce1 targeting strategy. Exons 2–11 (grey boxes) were replaced by LacZ-Neor selection cassette. Genotyping primers are marked by arrows (B) Southern blot analysis of DNA digested with EcoRI and hybridised with external probe (see A). A wild-type band of 11 kb is detected in the control and two bands 11 kb wild-type allele and 7.5 kb mutant allele in three clones, indicating correct targeting. (C) PCR genotyping using primers shown in (A). (D) Western blot analysis of testis cell extracts from wild-type and Syce1−/− mice. The blot was probed with anti-SYCE1 antibody. A protein of the correct size was detected only in the wild-type extract. Abbreviations: A - ApaI; E -EcoRI; S - SacI; Ex.Pr.- External Probe. (0.5 MB TIF) [file pgen.1000393.s002.tif]

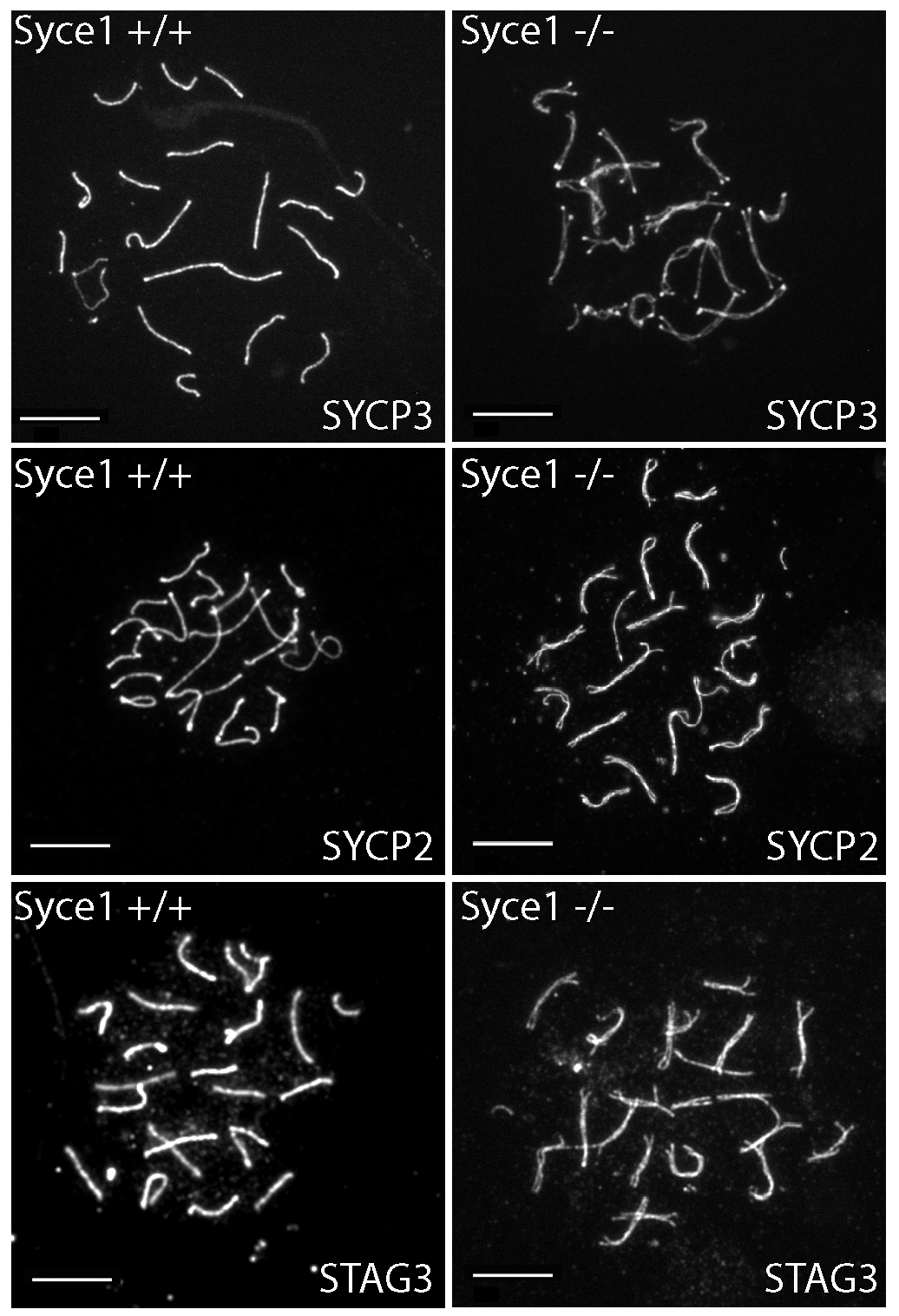

Supplement: Figure S3 — Syce1 mutant mice form normal AEs that align homologously. Surface-spread nuclei of wild-type and mutant meiotic cells were immunostained with antibodies against SC components SYCP2 and SYCP3 and cohesin STAG3. Scale bar 10 µm. (1.1 MB TIF) [file pgen.1000393.s003.tif]

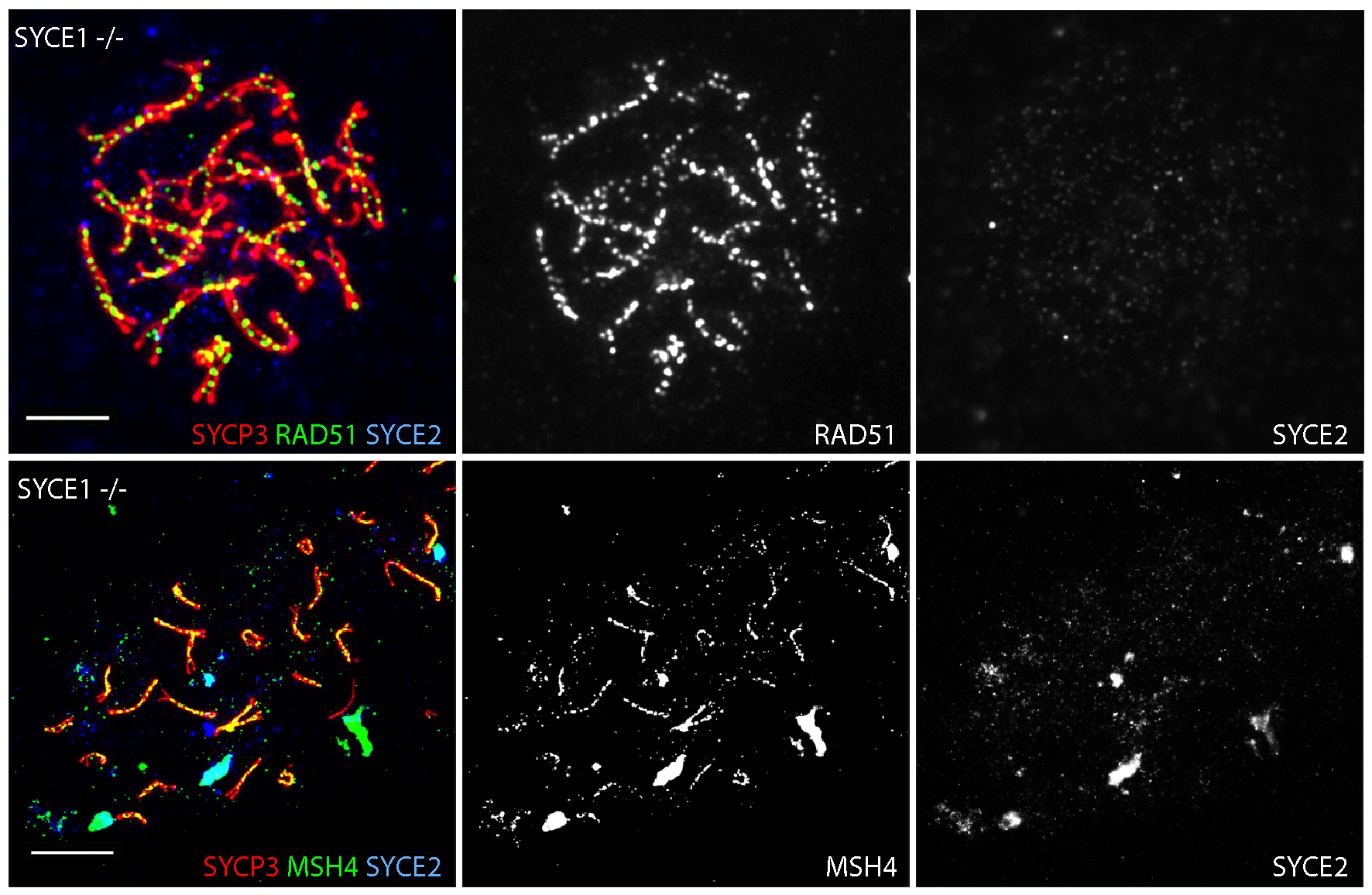

Supplement: Figure S4 — Immunostaining of representative Syce1−/− cells positive for RAD51 or MSH4 but lacking SYCE2 signal. Scale bar 10 µm. (1.5 MB TIF) [file pgen.1000393.s004.tif]
